# Exogenous Putrescine Alleviates Drought Stress by Altering Reactive Oxygen Species Scavenging and Biosynthesis of Polyamines in the Seedlings of Cabernet Sauvignon

**DOI:** 10.3389/fpls.2021.767992

**Published:** 2021-12-14

**Authors:** Jiaqi Zhao, Xuefei Wang, Xingbo Pan, Qianqian Jiang, Zhumei Xi

**Affiliations:** ^1^College of Enology, Northwest A&F University, Xianyang, China; ^2^Shaanxi Engineering Research Center for Viti-Viniculture, Xianyang, China

**Keywords:** putrescine, polyamines biosynthesis, Cabernet Sauvignon, drought, ROS

## Abstract

Climate change imposes intensive dry conditions in most grape-growing regions. Drought stress is one of the most devastating abiotic factors threatening grape growth, yield, and fruit quality. In this study, the alleviation effect of exogenous putrescine (Put) was evaluated using the seedlings of Cabernet Sauvignon (*Vitis vinifera* L.) subjected to drought stress. The phenotype, photosynthesis index, membrane injury index (MII), and antioxidant system, as well as the dynamic changes of endogenous polyamines (PAs) of grape seedlings, were monitored. Results showed that drought stress increased the MII, lipid peroxidation, and the contents of reactive oxygen species (ROS) (H_2_O_2_ and O_2_^–^), while it decreased the antioxidant enzyme activity and the net photosynthesis rate (Pn). However, the application of Put alleviated the effects of drought stress by altering ROS scavenging, enhancing the antioxidant system, and increasing the net Pn. Put distinctly increased the activity of superoxide dismutase (SOD), peroxidase (POD), and catalase (CAT), as well as the contents of ascorbic acid (AsA) and glutathione (GSH). Meanwhile, exogenous Put also promoted the metabolism of endogenous PAs by upregulating their synthetic genes. Our results confirmed that the exogenous application of Put can enhance the antioxidant capacity as well as alter the PA pool, which provides better drought tolerance for Cabernet Sauvignon seedlings.

## Introduction

Climate change facilitates drought events in most areas of the world, which leads to unfavorable growing conditions in grapevine production areas. Drought stress is one of the costliest natural hazards for grapevine growth, productivity, and fruit quality in viticulture (Xu et al., 2020). The change in precipitation and the resulting water depletion impedes plant homeostasis and elicits physiological and biochemical responses, such as accumulation of osmotic substances, stomata closure, and a parallel decline in net photosynthetic rate (Pn) (Kumar et al., 2008; Farooq et al., 2009b; Zhang et al., 2020b). Additionally, drought stress induces an excessive production of ROS and a reduction in antioxidant enzyme activity, which disrupts photosynthesis, increases lipid peroxidation, and destroys cell membrane structure (Farooq et al., 2009a; Miller et al., 2010; Ju et al., 2020). In recent studies, the use of plant growth regulators has become more common to enhance plant drought tolerance, which has been considered a simple and low-cost approach. Polyamines (PAs) have been previously reported to be involved in drought tolerance (Jahan et al., 2019a; Phornvillay et al., 2019; Seifikalhor et al., 2020; Zhang et al., 2020a); however, the relationship between putrescine (Put) and drought tolerance of wine grapes remains unclear.

Polyamines including Put, spermidine (Spd), and spermine (Spm) are a class of low-molecular-weight compounds, encompassing some of the metabolites that are ubiquitously present in plants (Wu et al., 2016; Yuan et al., 2019). In plants, arginine decarboxylase (ADC) and/or ornithine decarboxylase (ODC) converts arginine and ornithine to Put (Fortes et al., 2019). In plants, Spd and Spm are biosynthesized from Put and Spd through the successive addition of aminopropyl groups by the enzymes, Spd synthase (SPDS) and Spm synthase (SPMS) from decarboxylated *S*-adenosylmethionine (dc-SAM). *S-*adenosylmethionine decarboxylase (SAMDC) catalyzes *S-*adenosylmethionine (SAM) for dc-SAM synthesis. PAs are catabolized to 4-aminobutanal, 1,3-diaminopropane, and *N*-(3-aminopropyl)-4-aminobutanal by diamine oxidase (DAO) and polyamine oxidase (PAO) (Li et al., 2016).

Recent studies found that endogenous PAs markedly accumulated in rice, tobacco, and maize under drought stress (Capell et al., 2004; Bae et al., 2007; Alcazar et al., 2010; Li et al., 2018). The accumulation was upregulated by PA synthesis genes and the mutual transformation of free, conjugated, and bound forms of PAs (Shi et al., 2013a). Zhou and Yu (2010) found that vetiver grass accumulated more PAs to adapt to drought. Exogenous applications of PAs modulate drought responses in wheat through accumulating osmolytes, regulating the metabolism of Pas, and increasing free PAs (Ebeed et al., 2017). Hamid et al. (2018) had proved that Put decreased drought-induced ROS accumulation by increasing the activities of antioxidation enzymes (Hamid et al., 2018). Similar findings were also shown in maize, and the study proved that PAs enhanced the antioxidant defense system (Li et al., 2018). However, there are few reports of the effect of Put, an important precursor of PA synthesis, on the antioxidant defense system and endogenous PA homeostasis in wine grapes under drought stress.

The objective of this study was to investigate the effects of exogenous Put on enhancing drought tolerance of Cabernet Sauvignon (*Vitis vinifera* L.) seedlings. In addition to some basic physiological indicators, the network of the antioxidant system and how PA’s homeostasis contributed to drought resistance through exogenous application of Put were evaluated in grapevine for the first time. These results will improve our understanding of the relationship between Put and drought tolerance and provide a new strategy to enhance the plant’s drought tolerance.

## Materials and Methods

### Plant Materials, Growth Conditions, and Treatments

Hardwood cuttings of “Cabernet Sauvignon” (*Vitis vinifera* L.) were soaked in plant rooting hormones (20% naphthaleneacetic acid, 30% indole acetic acid) for 6 h, transferred to sand, and incubated on a heat bench at 24°C for root generation. The seedlings with roots were transferred into pots (20 cm × 16 cm) containing a mixture of soil, humus, and perlite (4:2:1, v/v). All seedlings were grown in a green house at 24 ± 1°C (day) and 16 ± 2°C (night) under 14-h light/10-h dark light cycles and 165 μmol • m^–2^• s^–1^ of light intensity. Uniformly grown grape seedlings with eight to ten fully expanded leaves were selected for Put and drought treatment. The pots were watered to soil water saturation, the day before Put treatment. All seedlings were randomly divided into four groups, and received foliar treatments as follows: CK, sterile water; T1, 0.01 mmol⋅L^–1^ Put; T2, 0.10 mmol⋅L^–1^ Put; T3, 1 mmol⋅L^–1^ Put. About 0.1% of Tween-80 was added to all Put solutions. All leaves were sprayed on both sides until dripped naturally. Thirty-five seedlings were used in each group. The day after Put pretreatment was set as Day 0 and irrigation was withheld for the following 6 days to reach drought stress. Soil water content was monitored daily with a TDR soil moisture meter (Spectrum Technologies, TX, United States). The first three leaves from the top were used for gene quantification and the fifth leaves of three seedlings were sampled daily and stored at −80°C for further analysis.

### Assessment of Drought Response and Photosynthetic Characteristics

All fully expanded leaves were used for drought stress evaluation based on the following visual rating grades of leaf characteristics (0–3): 0 = leaf was healthy, 1 = dehydration appeared on the leaf surface, 2 = leaves were dehydrated and yellow patches appeared on the leaves, and 3 = seedlings exhibited visual signs of drought damage, including yellow foliage, leaf drop, and plant wilt. The drought index was defined as Σ(leaves × grade)/(total leaves × the highest grade) × 100%.

Leaf gas exchange parameters, including the net photosynthetic rate (Pn), stomatal conductance (Gs), transient transpiration rate (Tr), and intercellular CO_2_ concentration (Ci), were measured daily between 9:00 and 11:00 am with an LI-6400 photosynthesis system (LI-COR Bioscience, Lincoln, NE, United States). Plants were monitored in a growth chamber with the light intensity of 1,500 μmol⋅m^–2^⋅s^–1^, the temperature of 30°C. External air was scrubbed of CO_2_ and mixed with pure CO_2_ to generate a concentration of 450 μl⋅L^–1^.

### Measurement of Membrane Injury Index and Lipid Peroxidation

The membrane injury index (MII) of leaves was quantified using a modification of the method described by Jahan et al. (2019b). Briefly, 30 leaf discs were cut with a 1 cm-circular puncher. The leaf discs were washed three times with ddH_2_O, vacuumed in ddH_2_O for 20 min. The initial electrical conductivity (T_1_) in the bathing solution was determined with a DDS-307A conductivity meter (LEICI, Huizhou, China). Subsequently, samples were boiled at 95°C for 20 min and final electrical conductivity (T_2_) was measured after the samples were cooled to room temperature. Analogously, the conductivity of deionized water was used as a control (C_1_ and C_2_). The MII was calculated as follows:


$$MII\left(\%\right)=\frac{T_{1}-C_{1}}{T_{2}-C_{2}}\times 100$$

Lipid peroxidation products were determined based on Malondialdehyde (MDA) content using the thiobarbituric acid reactive substances (TBARS) assay by Dhindsa et al. (1982) with modifications. About 0.5 g of leaf samples were homogenized in a 5.0 ml 10% (w/v) thiobarbituric acid (TBA) solution and centrifuged at 4°C for 20 min at 12,000 × *g*. A supernatant measuring 2.0 ml was mixed with 2.0 ml TBA, boiled at 100°C for 20 min, and cooled on ice. Subsequently, the mixture was centrifuged at 4,400 × *g* for 10 min. MDA content was measured at 450, 532, and 600 nm by the UV-1800 spectrophotometer (Shimadzu Corporation, Kyoto, Japan).

### Measurement of H_2_O_2_ Content and O_2_^⋅–^ Producing Rate

The content of H_2_O_2_ was determined using an H_2_O_2_ assay kit (Suzhou Keming Biotechnology Co., Ltd., Suzhou, China) following the instructions of the manufacturer. Briefly, 0.1 g of leave samples were homogenized with acetone in an ice bath and centrifuged at 8,000 × *g* for 10 min at 4°C. About 25 μl titanium sulfate and 50 μl ammonia were added to 250 μl of supernatant and then centrifuged at 4,000 × *g* for 10 min. The pellet was mixed with 1 ml of sulfuric acid. The absorbance was measured at 415 nm. The H_2_O_2_ content was calculated based on the standard curve and expressed as μmol g^–1^ FW.

The superoxide radicals (O_2_^⋅–^) producing rate was determined following the procedure reported by Chen et al. (2019). Briefly, 0.2 g leaves were homogenized with 2 mL of phosphate buffer solution (PBS, 50 mM, pH 7.8) and centrifuged at 12,000 × *g* for 20 min at 4°C. About 0.2 ml of PBS (50 mM), 0.2 mL of anhydrous sulfanilic acid (17 mmol⋅L^–1^), and 0.2 ml of α-naphthylamine (7 mmol⋅L^–1^) were successively added to 0.2 ml of supernatant and incubated at 30°C for 20 min. The absorbance was read at 530 nm. The O_2_^⋅–^ producing rate was calculated with a standard curve of NaNO_2_ and expressed as μg⋅g^–1^⋅min^–1^.

### Measurement of Antioxidant Enzymes and Metabolites

The activity levels of SOD, POD, CAT, ascorbate peroxidase (APX), glutathione reductase (GR), dehydroascorbate reductase (DHAR), and monodehydroascorbate reductase (MDHAR) were determined using the protocols described by Wang et al. (2019). Results were calculated and expressed as U⋅g^–1^⋅min^–1^.

Total AsA was assayed using the modified methods of Kampfenkel et al. (1995). About 0.5 g leaves were ground with a mortar and pestle in ice-cold 5% sulfosalicylic acid and then centrifuged at 15,000 × *g* for 20 min at 4°C. About 300 μl of leaf supernatant was transferred and mixed with the reaction solution (75 μl of 1.84 M triethanolamine, 600 μl of 2.5 mM EDTA, and 200 μl of 100 mM dithiothreitol). The mixture was water bathed at 25°C for 10 min, and then mixed with 600 μl of 10% acetocaustin, 600 μl of 44% phosphoric acid, 600 μl of 2% 2,2-dipyridine, 300 μl of 3% FeCl_3,_ and 200 μl of dH_2_O. The mixture was water bathed at 40°C for 40 min and then read at 525 nm. The content of GSH was determined using the Solarbio kit (Solarbio, Beijing, China) following the manufacturer’s instructions. About 0.1 ml of the leaf supernatant was mixed with 0.7 ml of PBS (pH 7.7) and 0.2 ml of dithionitrobenzoic acid (4 mM). The absorbance was measured at 412 nm with the UV-1800 spectrophotometer (Shimadzu Corporation, Kyoto, Japan). AsA and GSH contents were expressed as μg AsA⋅g^–1^ FW and nmol GSH⋅g^–1^ FW.

### Carbohydrate Measurement

About 1 g of ground freeze-dried leaf samples were supplemented with 5 ml of 80% ethanol for 30 min in a water-bath at 80°C and then centrifuged 3,500 × *g* for 10 min. The ethanol extracts were used for the quantification of the total soluble sugar, glucose, fructose, and sucrose, while the residues were further processed for starch extraction and analysis (Gomez et al., 2007). Glucose, fructose, and sucrose contents were quantified through enzymatic methods coupled to NADH production monitoring with a spectrophotometer at 340 nm. For starch quantification, the residues were washed with ddH_2_O and dried at 60°C for 30 min. About 500 μl of NaOH (0.5 M) was added to the dry residues and incubated at 60°C on a shaker for 1 h. After cooling, the mixture was neutralized with 25 μl of acetic acid (1M) and 475 μl of ddH_2_O. The mixture was vortexed at 4,200 × g for 10 min. About 10 μl of the mixture was transferred to a 96-well microplate and digested with amyloglucosidase (AG) and hexokinase/glucose 6-phosphate dehydrogenase (HK/G6P-DH) (Roche Applied Science, Penzberg, Germany). Absorbance was recorded at 340 nm. The calculations were based on the standard curve derived from glucose standards as described by Gomez et al. (2007).

### Endogenous Polyamines Derivation and Analysis

Endogenous free PAs were derived and analyzed by high-pressure liquid chromatography (HPLC) according to the method of Huang et al. (2017) with minor modifications. About 0.3 g of grape leaves were homogenized with 3 ml of 5% (w/v) cold perchloric acid. The homogenates were allowed to stand on ice for 1 h and then centrifuged at 23,000 × *g* for 30 min at 4°C. About 0.5 ml of the supernatant was mixed with 1 ml NaOH (2 mol⋅L^–1^) and 10 μl of benzoyl chloride and incubated for 20 min at 37°C. The mixture was added to 2 ml of the saturated NaCl solution and 2 ml of diethyl ether and centrifuged at 1,500 × *g* for 5 min at 4°C. The diethyl ether phase measuring 1 ml was collected, evaporated to dryness under a stream of warm air, and dissolved in 100 μl of methanol. The benzoyl-polyamines extract was filtered through a 0.22 μm membrane filter. HPLC (Agilent, CA, United States) was used to separate and analyze the content of the PAs with a C18 research-phase column at a flow rate of 1.0 mL⋅min^–1^. The mobile phases include methanol-water in the ratio of 60/40 (v/v), at a flow rate of 1.0 mL⋅min^–1^. The analyses of all PAs were detected with three biological replications.

### RNA Extraction and qRT-PCR

Total RNA was extracted from leaves using Universal Plant Total RNA extraction Kit (Bioteke Corporation, Beijing, China), following the manufacturer’s protocol. Total RNA measuring 400 pg was purified and reverse-transcribed using PrimeScript^®^RT reagent Kit with gDNA Eraser (TaKaRa, Dalian, China). The qRT-PCR analysis was performed using a QuantStudio™ 6 Flex Real-Time PCR System (Applied Biosystems, Foster City, CA, United States). All qRT-PCR assays were carried out in triplicate using a reaction volume of 25 μl. Each reaction contained 12.5 μl of SYBR Green dye (TaKaRa, Dalian, China), 5 μl of each primer, 1 μl of diluted cDNA, and 10.5 μl of RNA-free water. Primers used for the real-time PCR are shown in Supplementary Table 1. *VvActin* was used as the internal control. The relative gene expression was calculated by the 2^–ΔΔCt^ formula (Livak and Schmittgen, 2001).

### Statistical Analyses

All the data were statistically analyzed with SPSS 20.0 (SPSS Inc., Chicago, IL, United States). Mean comparisons were performed using standard ANOVA followed by Duncan’s multiple range test (*P* < 0.05).

## Results

### Effects of Exogenous Put on Seedling Growth and Physiological Response Under Drought Stress

**FIGURE 1:**
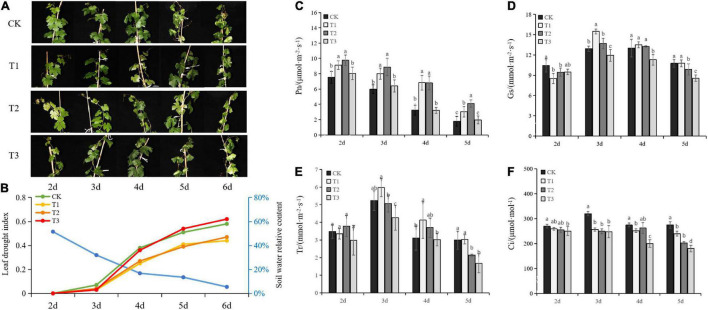
Effects of putrescine (Put) on seedling growth and physiological response under drought stress. Morphology of seedling growth **(A)**, Soil water relative content (blue line) and leaf drought index **(B)**, Net photosynthetic rate (Pn) **(C)**, stomatal conductance (Gs) **(D)**, transpiration rate (Tr) **(E)**, and internal carbon dioxide concentration **(F)**. T1: 0.01 mmol⋅L^– 1^ of Put, T2: 0.1 mmol⋅L^–1^ of Put, T3: 1 mmol⋅L^–1^ of Put, CK: water control. Each value is the mean of three replicates ± standard error (SE). Different letters above the error bars indicate significant difference at the 0.05 level according to Duncan’s multiple range test.

Leaf morphology, drought index, and the relative content of the soil water were monitored after withholding irrigation to evaluate the effects of Put on seeding growth under drought stress. The leaves started to dehydrate at Day 3 (Figure 1A), and the relative content of the soil water was decreased (Figure 1B). Under the continuous drought treatment, the relative content of water was notably decreased. Significant evidence of drought stress, such as yellow foliage and wilting, was observed on Day 6. The seedlings responded differently to the three Put concentrations. After Day 4, the seedlings treated with T1 and T2 maintained lower drought indices compared with those from control and T3 after Day 4. However, there was no difference observed between T3 and CK. This result suggested that lower Put concentrations alleviated the drought damage and facilitated the acclimation of plants to drought stress.

To gain insight into the effects of Put on physiological response, photosynthetic properties were investigated. Pn, Gs, Tr, and Ci decreased as drought stress was progressively established after Day 3.

The photosynthetic rate was significantly higher in the seedlings treated with Put compared with the control, except in T3 (Figure 1C). T1 and T2 treatments resulted in a slight stomatal opening at Day 3, while the Gs in T3 decreased significantly more in the control (Figure 1D). As shown in Figure 1E, T1 and T2 treatments slightly increased the Tr in the middle of drought treatment. Tr in T3 was significantly decreased due to stomatal closure compared to the control. With the intensified exposure to drought stress, the Ci of all treatments was slightly decreased after Day 3 (Figure 1F). Put-treated seedlings showed lower Ci than drought-stressed controls. These results indicated that the low levels of exogenous Put had a positive effect on photosynthesis stimulation, stomatal control, and the balance of CO_2_-uptake and water loss in the seedlings under drought stress.

### Membrane Injury and Oxidative Stress in Response to Exogenous Put Treatment

**FIGURE 2:**
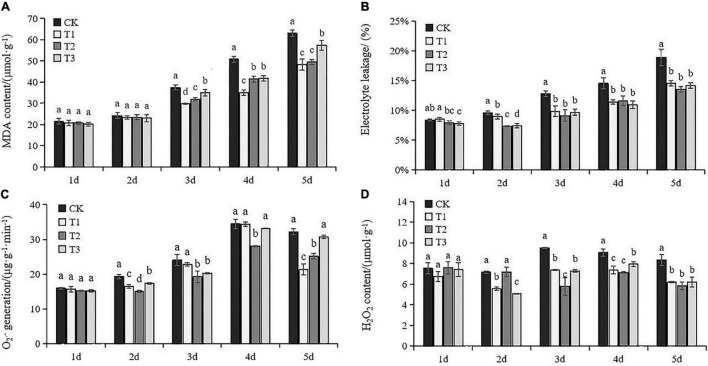
Membrane injury and oxidative stress in response to exogenous Put treatment. Malondialdehyde (MDA) **(A)**, electrolyte leakage **(B)**, O_2_^–^ producing rate **(C)**, H_2_O_2_ content **(D)**. Each value represents the mean of three replicates ± SE. Different letters above the error bars indicate significant differences at the 0.05 level according to Duncan’s multiple range test.

Damage to leaf cellular membranes was indicated by accumulated MDA and higher electrolyte leakage after Day 3. MDA content in the leaves with exogenous Put treatment was significantly lower than CK after Day 3 (Figure 2A). Application of lower Put concentrations (T1 and T2) was more effective for alleviating MDA accumulation under drought stress compared to T3. In addition, Put treatments significantly suppressed electrolyte leakage under drought stress compared to the results of untreated seedlings (Figure 2B). There was no obvious change in electrolyte leakage among different Put treatments after Day 3. Results indicated that Put prevented cell damages caused by drought stress.

The highly reactive superoxide anion is catalyzed to O_2_^⋅–^ and H_2_O_2_. The drought-induced oxidative stress was therefore addressed using the O_2_^⋅–^ producing rate and H_2_O_2_ accumulation. No significant difference was observed between the Put treatment groups and control at Day 1 (Figures 2C,D). Increases in O_2_^⋅–^ and H_2_O_2_ production were obvious in the control leaves subjected to drought stress. Application of Put led to a substantial reduction of H_2_O_2_ compared with the controls. T2 insistently led to sharp decreases in O_2_^⋅–^ and H_2_O_2_ production after Day 3. These results suggested that the effects of Put on grape drought tolerance might be related to the inhibition of ROS overproduction.

### Changes in Antioxidant Enzyme Activity and Antioxidant Substances

**FIGURE 3:**
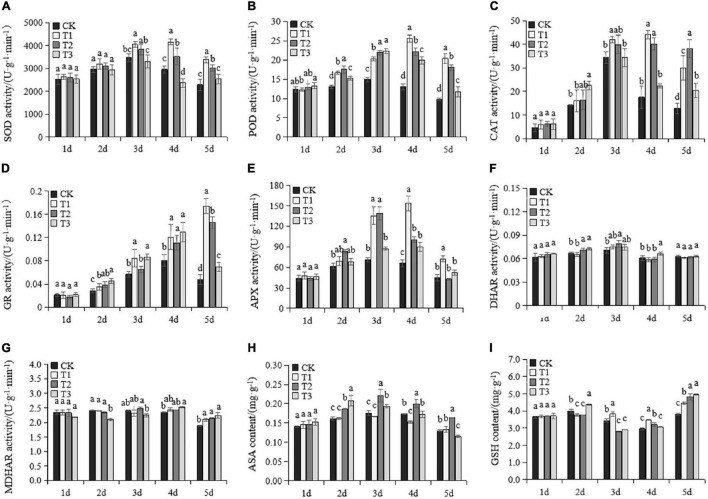
Changes of antioxidant enzyme activity and antioxidant substances in the seedlings of Cabernet Sauvignon under drought stress. The activity of superoxide [SOD, **(A)**], peroxidase [POD, **(B)**], catalase [CAT, **(C)**], glutathione reductase [GR, **(D)**], ascorbate peroxidase [APX, **(E)**], dehydroascorbate reductase [DHAR, **(F)**], and monodehydroascorbate reductase [MDHAR, **(G)**]. The contents of total ascorbic acid [AsA, **(H)**] and glutathione [GSH, **(I)**]. Each value represents the mean of three replicates ± SE. Different letters above the error bars indicate significant differences at the 0.05 level according to Duncan’s multiple range test.

To evaluate the remediation role of Put treatments in ROS stress, we investigated the antioxidant enzymes and non-enzymatic substances upon exposure to drought stress (Figure 3). All enzymes and antioxidant substances were comparable in all treatments at Day 1. The SOD, POD, CAT, and APX activity, as well as AsA content, increased rapidly as the drought established and gradually decreased after Day 4. All Put treatments consistently induced POD activity under drought stress compared to the results in CK. SOD and CAT in T1 were significantly induced on Day 3. After Day 4, the activities of the two enzymes increased significantly in both T1 and T2 compared to the activities in CK and T3. The activity of CAT in T3 only increased notably on Day 5, while SOD levels in T3 were depressed at Days 3 and 4. GR activity of all treatments gradually increased under drought stress, except in CK and T3, which decreased on the fifth day (Figure 3D). Exogenous Put treatments increased GR activity compared to the activity in CK on the second, third, fourth, and fifth days under drought stress. On the fifth day, the GR activity of T1 and T2 treatments reached the maximum, which were 3.6 times and 3.1 times higher than those of CK, respectively. APX activity in T1 was consistently higher after Day 3 (Figure 3E). APX was highly induced by T2 on Day 2 and Day 3 but dramatically declined over the final 2 days. DHAR and MDHAR activity remained stable after drought treatments, with slight fluctuations (Figures 3F,G). Put treatments caused significant rises in MDHAR activity on the fifth day of drought stress. AsA and GSH are two common antioxidants in plants. The level of AsA in T1 was like that in the control (Figure 3H). AsA was not degraded in T2 and maintained its highest levels after Day 3. First, the GSH content decreased and then increased under drought stress. The GSH content in T1 was significantly higher than CK after Day 3. GSH started to accumulate in T2 after Day 4. The GSH content in T3 was significantly lower than CK at early drought stages and later was significantly amplified on Day 5. Put treatments had no effects on the antioxidant enzymes and non-enzymatic substances on Day 1. After this, T1 and T2 significantly accelerated the antioxidant system, which helped maintain lower ROS stress than the other treatments.

### The Content of Soluble Sugar and Starch

**FIGURE 4:**
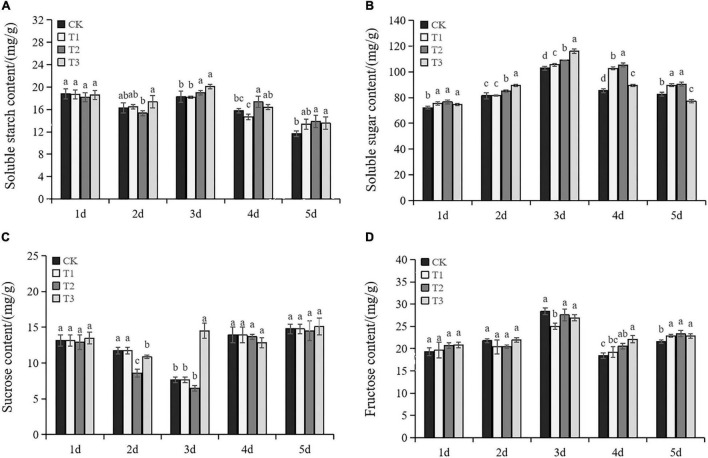
Changes of soluble starch content **(A)**, soluble content **(B)**, sucrose content **(C),** and fructose content **(D)** in the seedlings of Cabernet Sauvignon under drought stress. Each value represents the mean of three replicates ± SE (standard error) shown by vertical error bars. Different letters above the bars indicate significant differences at the 0.05 level according to Duncan’s multiple range test.

As shown in Figure 4, soluble starch, soluble sugar, and fructose showed trends of increasing and then decreasing in exogenous Put treatments. The soluble starch content in CK gradually declined, while T2 and T3 were higher than CK under drought stress (Figure 4A). Exogenous Put significantly increased soluble sugar content compared to CK, except T3 on the fifth day (Figure 4B). The sucrose content gradually decreased before Day 3 and increased significantly after Day 4 (Figure 4C). Put presented different effects on sucrose. No significant difference was observed between T1 and CK. The sucrose content of T2 and T3 at Day 2 was lower than CK. However, T3 significantly increased the sucrose content on Day 3. There was no significant difference in the content of fructose in each treatment before Day 3, except T2 on Day 3. Nevertheless, exogenous Put significantly increased the fructose content on Days 4 and 5. This evidence suggests that exogenous Put can increase the accumulation of osmotic substances under drought stress.

### The Changes of Endogenous Polyamines and Gene Expression of Polyamine Anabolism Pathway

**FIGURE 5:**
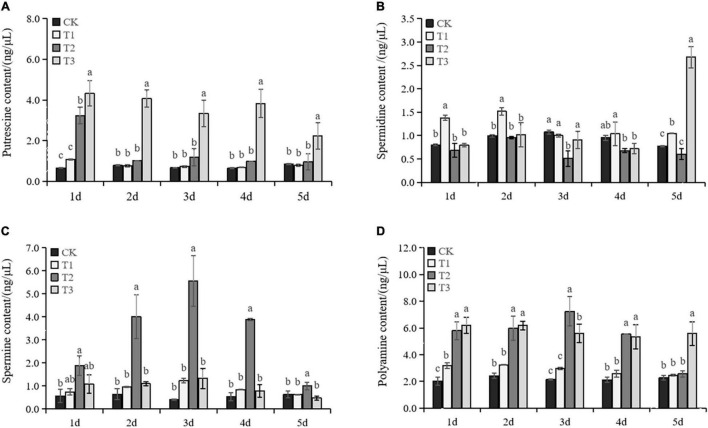
Changes in the content of Put **(A)**, Spd **(B)**, Spm **(C)**, and PA **(D)** in the seedlings of Cabernet Sauvignon under drought stress. Each value represents the mean of three replicates ± SE (standard error) shown by a vertical error bar. Different letters above the bars indicate significant differences at the 0.05 level according to Duncan’s multiple range test.

Total PAs significantly increased when exogenous Put was applied (Figure 5D). PAs declined in T1 at Day 2, but consistently maintained a high level in T3 until Day 5. The Put content was slightly increased in T1 and T2 treatments under drought (Figure 5A). In T3 treatment, the Put content was significantly higher than in CK. The Spd content in T1 was significantly increased compared with the content in CK. T2 showed decreased Spd content in grape leaves under drought stress. In T3, the Spd content in T3 showed no significant change over the first 4 days but then increased dramatically at day 5 (Figure 5B). The Spm content was not affected significantly by T1 and T3 treatments under drought. However, T2 showed a significant boost in Spm accumulation under drought stress and it peaked on the third day of the drought (Figure 5C). The results showed that exogenous Put could stimulate the accumulation of endogenous PAs.

**FIGURE 6:**
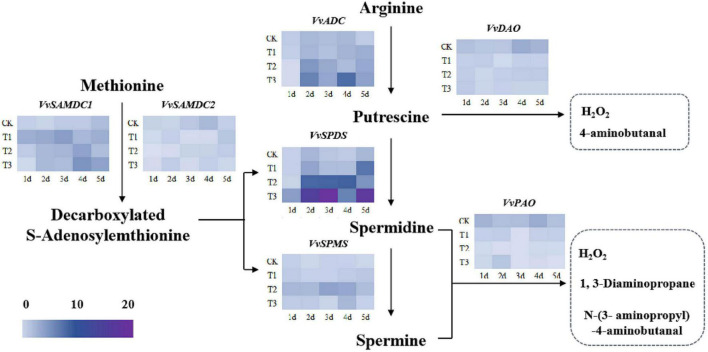
Gene expressions of polyamine (PA) anabolic pathways in the seedlings of Cabernet Sauvignon under drought stress, including *VvADC*, *VvSPDS*, *VvSPMS*, *VvSAMDC1*, *VvSAMDC2*, *VvDAO*, and *VvPAO.* The blue to purple scale represents low to high levels of expression respectively.

The expression levels of various genes involved in PA synthesis and catabolite pathways were analyzed in the grape leaves under drought stress. As shown in Figure 6, PA biosynthesis genes include *VvADC*, *VvSPDS*, *VvSPMS, VvSAMDC1*, and *VvSAMDC2*. The results showed that the expressions of *VvADC*, *VvSPDS*, and *VvSPMS* were upregulated under drought stress after the stimulation of exogenous Put. Notably, an abundance of the *VvSPDS* gene was noted in T2 and T3 and was significantly higher under drought stress in those groups than in CK. *VvSAMDC1* and *VvSAMDC2* are also key genes in a PA synthetic pathway. The results showed that exogenous Put played a pivotal role in upregulating the expression of *VvSAMDC1* under drought stress. However, the expression of *VvSAMDC2* in T1, T2, and T3 showed no significant change from CK. Additionally, in the PA metabolic pathway, exogenous Put downregulated the expression of *VvDAO* and *VvPAO*, which led to the inactivation of the corresponding enzymes and decreased H_2_O_2_ in response to drought stress.

## Discussion

Climate change increases the potential for higher drought stress during the growing season (Santillán et al., 2019). Identifying measures to alleviate drought stress in grapevine is an urgent concern for both grape growers and wine producers. PAs play diverse roles in responses to drought stress stimulation in plants (Zhang et al., 2018; Doneva et al., 2021). The effect of exogenously applied Put and its drought tolerance mechanisms were investigated and confirmed in the current as well as the previous study, where the PAs and redox homeostasis mitigated environmental stresses (Li et al., 2018).

### Put Increased Plant Photosynthesis and Accumulation of Osmotic Substances

Photosynthetic abnormalities and the appearance of osmotic substances are typical symptoms that are present when plants suffer from water stress (Farooq et al., 2009b). Stomatal conductance decreased after Day 3 under drought stress, which resulted in the decrease of Pn and Tr. Nevertheless, the stomatal opening was increased under low concentrations of Put treatments compared to the results in the control which was consistent with the study on wheat (Szalai et al., 2017). Stomatal movement is a critical trait controlling water transpiration and CO_2_ absorption, especially under drought stress (Berahim et al., 2021). Tr increased accordingly with the increased stomatal opening. The increase of Pn in Put treatments indicated that Put may increase the plant photosynthesis under drought stress through non-stomatal factors in chloroplasts and interact with thylakoid membranes (Hamdani et al., 2011; Wu et al., 2019). Furthermore, decreased Ci was attributed to increased CO_2_ assimilation after Put treatments, which provides additional insight into plant growth and development (Szalai et al., 2017).

Exogenous Put significantly increased soluble sugar accumulation under drought stress, which is closely related to a signaling adjustment to environmental response. It is likely because Put is polycationic, meaning it can bind and stabilize osmotic micromolecules (Qu et al., 2020). Sucrose metabolism is often used as an indicator of environmental stress and plant adaptability. Under drought stress, exogenous Put slightly increased the content of fructose after Day 4 under drought but had no effect on the content of sucrose. This demonstrated that Put may promote more fructose accumulation and can better maintain osmotic balance and water potential. Put also significantly increased soluble starch content under drought stress which may be related to the increased net Pn of leaves. This evidence suggested that exogenous Put can increase drought tolerance by accumulating sufficient osmotic regulators.

### Putrescine Balanced ROS Homeostasis in Grape Seedlings Under Drought Stress

Drought stress leads to the overproduction of ROS in plants, causing damage to lipids, carbohydrates, proteins, and DNA, which results in oxidative stress, toxin production, reduction of cell membrane stability (CMS), and ultimately cell death (Apel and Hirt, 2004; Miller et al., 2010; Wang et al., 2019; Zhu et al., 2020). The antioxidant defense system for scavenging toxic ROS is stimulated by exogenous Put, including non-enzymatic and enzymatic components. In the present study, the higher enzyme activity of SOD, CAT, and POD was found in Put treatments. Similar results of higher POD isozymes activity were reported in wheat in the previous studies (Pál et al., 2018a). SOD converts O_2_^⋅–^ to H_2_O_2_ and gives frontline protection against ROS. Subsequently, CAT and APX scavenge H_2_O_2_ to H_2_O (Hasanuzzaman et al., 2019). Exogenous Put distinctly decreased the electrolyte leakage and the contents of ROS under drought stress. Similar results were also found in the study of trifoliate orange (Wu et al., 2016). The results agreed with the previous study that showed PAs modulated the stress-triggered ROS production and oxidative damage by activating the antioxidant enzyme activities (Shi et al., 2013a,b). Previous evidence suggests that PAs conjugates can directly bind and remove ROS (Hussain et al., 2011).

The AsA-GSH cycle also plays an important role in maintaining ROS homeostasis (Miller et al., 2010). AsA and GSH participated in the AsA-GSH cycle to maintain ROS homeostasis, as well as scavenge ROS directly (Foyer and Noctor, 2011). Exogenous Put increased the activity of MDHAR at late drought stress, which promoted the transformation of monodehydroascorbate (MDHA) to AsA. At the same time, the activity of APX increased significantly after the third day of exogenous Put stimulation. APX converts H_2_O_2_ to H_2_O with AsA as an electron donor (Park et al., 2016). GR activity was also significantly increased under the stimulation of exogenous Put, which led to the accumulation of GSH in the late stage of drought stress. High production of AsA and GSH enhances the ability of plants to resist oxidative stress. These results were consistent with the previous studies showing that exogenous PAs could maintain redox homeostasis by generating GSH and AsA content *via* upregulation of the enzymatic components of the AsA-GSH cycle (Gill et al., 2013; Mohammad et al., 2019). Moreover, exogenous Put promotes the AsA-GSH cycle due to the upregulation of AsA-GSH synthesis-related genes at the transcriptional level (Li et al., 2019). All results indicated that exogenous Put reduced oxidative stress by increasing the antioxidant enzymes and regulating the AsA-GSH cycle, which further alleviated the oxidative stress and enhanced drought tolerance of the grapevine.

### Put Induced the Biosynthesis of Endogenous Polyamines

Accumulation of PAs is a common stress response in plants (Liu et al., 2015; Wu et al., 2016; Zhang et al., 2020a). Nayyar and Chander (2010) found drought-sensitive chickpeas accumulated more PAs than drought-tolerant ones. Put, Spd, and Spm play distinct roles in drought resistance. It has been confirmed that exogenous Put affected the synthesis of endogenous Put and Spm among the three PAs by upregulating PA synthetic genes and downregulating decomposed genes (Ebeed et al., 2017; Pál et al., 2018b). PA conversion, but not *de novo* biosynthesis, occurred in grape seedlings when they were subjected to drought stress. *VvSPD* expression increased significantly while Spd content decreased, which maximized Spm synthesis. Spm is involved in abscisic acid-dependent pathways, signaling transduction, root growth, and development, which in turn protected plants from drought under water deficit conditions (Hasan et al., 2021).

In the present study, the endogenous accumulation of PAs was closely related to the ROS scavenging by the activation of antioxidant enzymes under abiotic stresses and scavenging of the free radicals (Gupta et al., 2013; Li et al., 2019). Due to the removal of excess ROS, the integrity of the leaf cellular membrane ensured photosynthesis under drought stress. It was also reported that Spd and Spm at high concentrations are efficient uncouplers of photophosphorylation (Duan et al., 2008). Furthermore, the higher polycationic character of the amine being used is accompanied by higher effectiveness in PSII efficiency restoration (Ioannidis and Kotzabasis, 2007; Gill and Tuteja, 2010). The accumulation of PAs led to the increase of net photosynthesis and the accumulation of osmotic substances in grape leaves under drought stress. Moreover, while three concentrations of Put were used in this study, the results showed that a low concentration of Put was more beneficial to the grape seedlings under drought stress. At high concentrations, Put produced a negative effect on the photosynthesis of grape seedlings. Similarly, Szalai et al. (2017) found that high concentrations of PAs inhibited the growth of maize, but this negative effect was not shown in wheat. This may be due to further synthesis, translocation, or other metabolic mechanisms of PAs in different species (Kubiś et al., 2014; Pál et al., 2018a). The results in this study demonstrated how the application of exogenous Put effectively reduced drought-related oxidative damage by the network of ROS scavenging and the metabolic accumulation of PAs.

## Conclusion

Exogenous Put significantly scavenged ROS as an antioxidative molecule by increasing the activity of antioxidant enzymes and accelerating the AsA-GSH cycle under drought stress. The protective effect was related to the concentration of Put. Excessive application of Put may lead to a negative effect resulting from excess PAs. The low levels of exogenous Put also increased net photosynthesis, affected the balance of CO_2_-uptake and water loss, and led to an accumulation of more osmotic regulators when the grape seedlings encountered drought stress. Moreover, Put also regulated the accumulation of endogenous PAs in grape leaves through the metabolism of PAs. Our results provided evidence of the positive effect of exogenous Put in enhancing drought tolerance of Cabernet Sauvignon seedlings. Nevertheless, the regulation mechanisms of Put-mediated PAs and redox homeostasis are still unclear, and future studies focusing on the manipulation of PA’s metabolism are vital to cope with the challenges of climate change and drought events.

## Data Availability Statement

The original contributions presented in the study are included in the article/Supplementary Material, further inquiries can be directed to the corresponding author/s.

## Author Contributions

JZ, XP, and QJ carried out the experiment and participated in data analysis. JZ, XW, and ZX designed the experiment, analyzed the data, and drafted the manuscript. All authors have revised this manuscript critically before the submission and agreed with all aspects of the study.

## Conflict of Interest

The authors declare that the research was conducted in the absence of any commercial or financial relationships that could be construed as a potential conflict of interest.

## Publisher’s Note

All claims expressed in this article are solely those of the authors and do not necessarily represent those of their affiliated organizations, or those of the publisher, the editors and the reviewers. Any product that may be evaluated in this article, or claim that may be made by its manufacturer, is not guaranteed or endorsed by the publisher.
