# Supplementary material for: Exogenous Putrescine Alleviates Drought Stress by Altering Reactive Oxygen Species Scavenging and Biosynthesis of Polyamines in the Seedlings of Cabernet Sauvignon
Source: Front Plant Sci. 2021 Dec 14;12:767992. doi: 10.3389/fpls.2021.767992 (PMC8712750; doi:10.3389/fpls.2021.767992)
Supplement: Supplementary file 1 [file Table_1.DOCX]

Supplementary Table 1. Primer pairs used in this study

| Gene ID | Gene name | Sequence (5’-3’) | Reference |
| --- | --- | --- | --- |
| VIT_03s0038g00760 | *VvADC* | F: CATCGTGGAAGATGTCGTGAA  R: ACCAGCTTCGAGGCCAAAC | Agudelo-Romero et al.(2013) |
| VIT_01s0026g00240 | *VvSPDS* | F: CTCTTTCACTGTCCTGAAACCATG  R: CAACCGCTGACCCCATTCT | Agudelo-Romero et al.(2013) |
| VIT_05s0020g03200 | *VvSPMS* | F: TGTGGCTTATTCAGCCCATATTC  R: AATGGCGAATCTGATGCCATA | Agudelo-Romero et al.(2013) |
| VIT_01s0010g00990 | *VvSAMDC1* | F: TTCCAACCGAATGAGTTCTCTGTAG  R: TCGCTCAAGTAACTTACCCGAGA | Agudelo-Romero et al.(2013) |
| VIT_11s0037g00950 | *VvSAMDC2* | F: ATACGCGTGTCAAAATGTGGTG  R: TTCATATGTCACGTACACCACGC | Agudelo-Romero et al. (2013) |
| VIT_17s0000g09100 | *VvDAO* | F: CTTTCGTGTACATGGGCACTATGT  R: ACATATGCAACAGAATGGATAACCAA | Agudelo-Romero et al. (2013) |
| VIT_04s0043g00220 | *VvPAO* | F: AAGTTCTGCTTGTGGTCTTGAGAAG  R: GGCTTAGTTGCATCAGGAAACATT | Agudelo-Romero et al. (2013) |
| NM001281024 | *VvActin* | F: GCACCCTTCGCACGATATGA  R: TGACGCAAGGCAAGGACTGA | Chen ZY et al. (2019) |
